# Anxiety and sleep quality in patients receiving maintenance hemodialysis: multiple mediating roles of hope and family function

**DOI:** 10.1038/s41598-024-65901-9

**Published:** 2024-07-02

**Authors:** Guoqing Wang, Xiang Yi, Hui Fan, Huiling Cheng

**Affiliations:** 1 Hemodialysis Center, Renmin Hospital of Wuhan University, Wuhan, 430060 Hubei China; 2 Department of Organ Transplantation, Renmin Hospital of Wuhan University, Wuhan, 430060 Hubei China; 3 Department of Respiratory and Critical Care Medicine, Renmin Hospital of Wuhan University, Wuhan, 430060 Hubei China

**Keywords:** Renal dialysis, Anxiety, Sleep quality, Hope, Family support, Mediating effect, Psychology, Nephrology

## Abstract

The objective of this cross-sectional study was to examine the extent of sleep quality among individuals undergoing maintenance hemodialysis (MHD) and to scrutinize whether hope and family function serve as mediators in the association between anxiety and sleep quality in this cohort. A convenience sampling method was used to recruit 227 patients receiving maintenance hemodialysis from two tertiary hospitals in Wuhan. Participants completed several self-report questionnaires, including the Sociodemographic questionnaire, Hospital Anxiety and Depression Scale, Athens Insomnia Scale, Herth Hope Index, and Family APGAR Index. As per the findings of the chain mediation analysis, it was observed that the sleep quality scores were directly predicted by anxiety. Moreover, anxiety positively predicted sleep quality scores through hope and family function as mediators. The observed types of mediation were partial mediation. The total indirect effect value was 0.354, indicating the mediating effect of hope and family function, while the total effect value was 0.481, representing the overall effect of anxiety on sleep quality. The total effect size was 73.60% (0.354/0.481), indicating that the mediation accounted for a significant portion of the relationship. This study established the chain mediating effect of hope and family function between anxiety and sleep quality in patients receiving maintenance hemodialysis. The findings highlight the importance of addressing anxiety and promoting hope and family function to improve sleep quality in this population. The findings suggest that healthcare professionals should be attentive to the anxiety levels of these patients and implement targeted interventions to help alleviate anxiety, enhance hope, and improve family functioning, with the ultimate goal of improving sleep quality in this population.

## Introduction

With the global population aging, the incidence of Chronic Kidney Disease (CKD) has been steadily increasing, making it a significant global public health concern. The projected overall prevalence of disease varies between 11.7% to 15.1%, and the mean count of individuals necessitating renal replacement therapy for End-Stage Renal Disease (ERSD) ranges from 4,902 to 7,803 per million population^1,2^. In China, the prevalence rate of CKD is 10.8%, and approximately 2% of patients progress to ESRD each year, with Maintenance Hemodialysis (MHD) being the preferred treatment modality. According to the CKD-NET report, the number of patients receiving hemodialysis has reached nearly 700,000^3^.

However, patients undergoing MHD often experience various comorbidities, including sleep disorders, particularly insomnia, which are common problems in this population^4,5^. Studies have shown that the incidence of sleep disorders in MHD patients ranges from 41 to 85%^6,7^. These sleep disorders are closely associated with inflammation and inflammatory diseases, such as infections, diabetes, cardiovascular diseases, and autoimmune diseases, and they significantly impact the patients' quality of life and mortality rates^8^.

The prolonged hemodialysis treatment and the burden of illness can lead to significant emotional distress in patients. Anxiety is the most common adverse emotional reaction, with an incidence of 40.0% to 53.4% in MHD patients^9^. The study has shown a negative correlation between anxiety and sleep quality, indicating that anxiety is one of the factors contributing to the decline in sleep quality and the development of sleep disorders in these patients^10^.

The hope theory model suggests that hope is the inner motivation and strong belief that individuals have in achieving their life goals^11^. The study has shown that hope is closely related to sleep quality, and individuals with higher levels of hope tend to have better sleep quality^12^. Family system theory emphasizes the role of the family as the primary social support system, providing practical care, companionship, and emotional communication. Good family functioning has been associated with improved sleep quality in patients^13^.

However, the current body of literature offers scarce insights into the association between anxiety, hope, family function, and sleep quality, particularly in the context of the MHD population. Therefore, this study aims to explore and analyze the potential mechanisms underlying sleep quality by examining the relationships between anxiety, hope, family function, and sleep quality. A structural Equation Modeling (SEM) will be used to evaluate the proposed hypotheses. SEM is a statistical methodology that takes a confirmatory (i.e.,hypothesis-testing) approach to the analysis of a structural theory bearing on some phenomenon^14^. Typically,this theory represents "causal" processes that generate observations on multiple variables. The findings from this study will contribute to future research on improving sleep quality in MHD patients and provide valuable insights for nursing practice. Improving sleep quality also reduces symptom severity and provides symptom management, thus increasing the quality of life.

The theoretical framework of this study is stress theory^15^. According to the psychological model of stress theory, stress is the mediating variable between a stressor and stress outcome, and cognitive evaluation and social support are the important mediating variables between a stressor and stress outcome. In this study, the relationship between hope and cognitive evaluation is that hope is an individual´s positive cognitive evaluation of the future. The relationship between family function and social support is that family function is an important part of social support. In this study, the high burden of economy, life and disease can be considered as traumatic events for MHD patients, which will lead to anxiety. Therefore, based on the previous literature review, this study concludes that hope (cognitive evaluation) and family function (social support) are mediating variables from anxiety (stress) to sleep disorders (stress outcome). Furthermore, cognitive evaluation (hope) can determine social support (family function), and there is an empirical correlation between hope and family function. Therefore, it can be concluded that there is a relationship between the two mediating variables.

**Figure 1:**
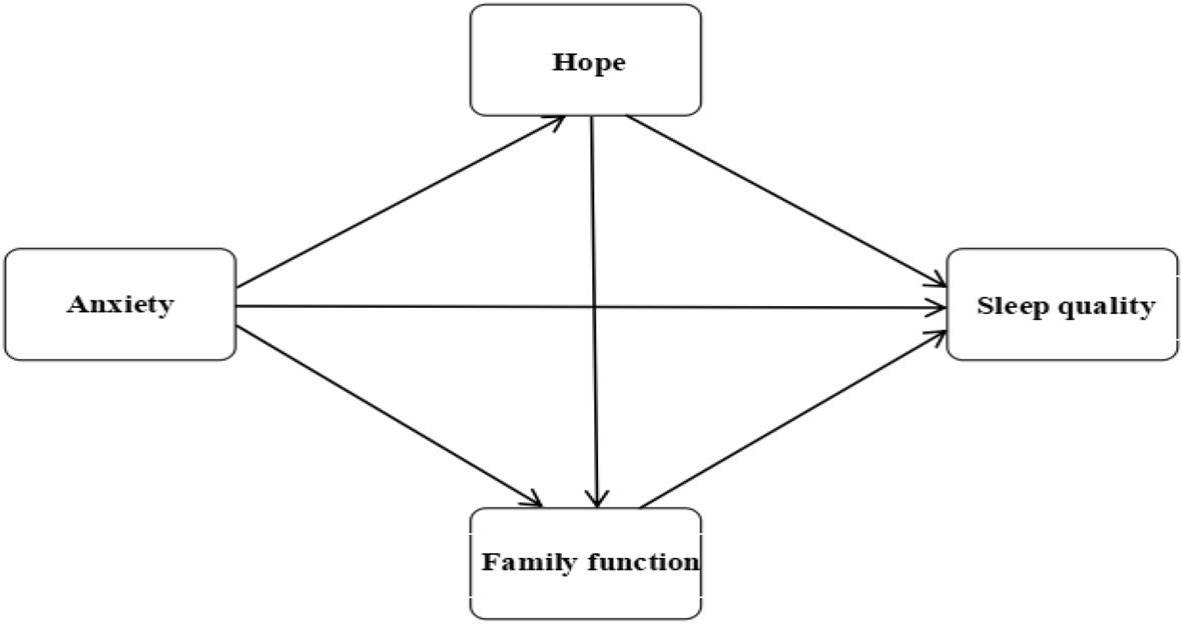
Concept diagram for the chain mediating effect model.

Based on the literature review and theoretical framework, it was hypothesized in this study that anxiety could affect the sleep quality of MHD patients through the chain mediation of hope and family function. Thus, four hypotheses were established and a concept diagram for the chain mediating effect model is shown in Fig. 1.

*H1*: Anxiety significantly predicts sleep quality (Anxiety → Sleep quality);

*H2*: Hope mediates the relationship between anxiety and sleep quality (Anxiety → Hope → Sleep quality);

*H3*: Family function mediates the relationship between anxiety and sleep quality (Anxiety → Family function → Sleep quality); and.

*H4*: Hope and family function mediates the relationship between anxiety and sleep quality (Anxiety → Hope → Family function → Sleep quality).

## Methods

### Design and sample

A descriptive cross-sectional survey was conducted to collect data from MHD patients, following the guidelines outlined in the STROBE statement. All methods were performed in accordance with the relevant guidelines and regulations. The study enrolled a total of 227 MHD patients from two tertiary hospitals in Wuhan, Hubei Province, China, between June and September 2021. The inclusion criteria for participants were as follows: (1) patients aged over 18 years old, (2) patients undergoing MHD treatment for more than three months, (3) patients who were conscious and able to independently complete the questionnaire or with the assistance of the researcher, and (4) patients who provided informed consent and willingly agreed to participate in the survey. The exclusion criteria included: (1) patients with pre-existing cognitive impairment, mental or psychological disorders, (2) patients with severe heart, liver, lung, or brain diseases, and (3) patients who had received psychological counseling within the three months prior to the study. A total of 250 questionnaires were distributed to MHD patients, of which 227 were deemed valid, resulting in an effective response rate of 90.8%.

In SEM, it is recommended to have a sample size that is approximately 10 to 15 times larger than the number of observed variables that are incorporated into the model^14^. Considering the observed variables in the study, including anxiety, sleep quality, adaptation, partnership, growth, affection, resolve, and the three factors of hope, a sample size of 100 to 150 would be appropriate. Taking into account the possibility of 20% invalid questionnaires, the desired sample size would be 120 to 180 participants. Thus, the inclusion of 227 participants in the study exceeds the required sample size, satisfying the criteria for the SEM analysis.

### Instruments

#### Sociodemographic characteristics

The Sociodemographic questionnaire was designed by the researchers after reviewing the literature^7–10^. The sociodemographic survey addressed gender, age, education, marital status, duration of dialysis, type of vascular access and diabetes in patients receiving MHD.

#### Hospital anxiety and depression scale (HADS)

The HADS developed by Zigmond and Snaith^16^ was utilized in this study. Specifically, the anxiety subscale of the HADS was employed. This subscale consists of 7 items, each rated on a four-point Likert scale ranging from 0 (definitely the same) to 3 (not at all). The total score on the anxiety subscale ranges from 0 to 21, with higher scores indicating a higher level of anxiety. The score interpretation for the anxiety subscale is as follows: scores ranging from 0 to 7 were classified as low level, scores ranging from 8 to 10 were labeled as medium level, and scores ranging from 11 to 21 were indicative of a high level of anxiety. The HADS anxiety subscale has been widely used among Chinese hemodialysis patients and has demonstrated effectiveness and reliability^17^. In this study, the Cronbach's alpha coefficient for the anxiety subscale was calculated to be 0.803, indicating a good level of internal consistency.

#### Athens insomnia scale (AIS)

The AIS developed by Soldatos et al.^18^ is a widely recognized self-report scale used to assess sleep quality. The instrument employed in this study evaluates an individual´s sleep quality during the preceding month. It comprises eight items, where in each item is rated on a four-point Likert scale that ranges from 0 (no difficulty) to 3 (significant delay or complete inability to sleep). The overall score on the aforementioned scale varies between 0 to 24, whereby higher scores indicate a poorer level of sleep quality. The score interpretation for the scale is as follows: scores ranging from 0 to 3 signify the absence of a sleep disorder, scores ranging from 4 to 6 suggest a potential for insomnia, and scores ranging from 7 to 24 indicate the presence of insomnia. The AIS has been used in previous study involving Chinese hemodialysis patients and has demonstrated effectiveness and reliability^19^. In this study, the Cronbach's alpha coefficient for the AIS was calculated to be 0.868, indicating a good level of internal consistency.

#### Herth hope index (HHI)

The HHI was developed by Herth^20^ to measure hope among individuals. It consists of 12 items that assess three factors: Factor 1 (temporality and future), Factor 2 (positive readiness and expectancy), and Factor 3 (interconnectedness). Each individual item is scored on a four-point Likert scale that ranges from 1 (strongly disagree) to 4 (strongly agree). The total score on the HHI ranges from 12 to 48, whereby higher scores indicate a greater level of hope. The score interpretation for the HHI is as follows: scores ranging from 12 to 23 indicate a low level of hope, scores ranging from 24 to 35 suggest a medium level of hope, and scores ranging from 36 to 48 indicate a high level of hope. The HHI has been used in previous study involving Chinese hemodialysis patients and has been shown to be effective and reliable^21^. In this study, the Cronbach's alpha coefficient for the HHI was calculated to be 0.870, indicating good internal consistency.

#### Family APGAR index

The Family APGAR Index was developed by Smilkstein^22^ to evaluate family function and consists of five items that are intended to measure five distinct dimensions of family function: Adaptation, Partnership, Growth, Affection, and Resolve. Each item is assessed on a three-point Likert scale that ranges from 0 (infrequent) to 2 (frequent). The total score on the Family APGAR Index ranges from 0 to 10, with higher scores indicating a higher level of family function. The score interpretation for the Family APGAR Index is as follows: scores ranging from 0 to 3 indicate a low level of family function, scores ranging from 4 to 6 suggest a medium level of family function, and scores ranging from 7 to 10 indicate a high level of family function. The Family APGAR Index has been used in previous study involving Chinese hemodialysis patients and has been shown to be effective and reliable^23^. In this study, the Cronbach's alpha coefficient for the Family APGAR Index was calculated to be 0.894, indicating good internal consistency.

### Data collection

The study was conducted while patients were undergoing MHD treatment. Participants were recruited from hemodialysis centers in two tertiary hospitals. Prior to data collection, the researchers received training to ensure they were familiar with the standardized measurement procedures and criteria for properly completed questionnaires. Participants were provided with a link to the questionnaire through WeChat, a popular communication software in China. The researcher connected with the participants on WeChat (participants were added to the WeChat contact list), so that the participants could submit both the link and their responses directly through WeChat. The purpose of the study, informed consent process, and instructions for completing the questionnaire were explained on the first page. Participants were given the option to independently complete the questionnaire or seek guidance from the researcher. The researcher promptly checked the submitted questionnaires and addressed any incomplete ones on the spot. The implementation method was that the researcher immediately reviewed the questionnaire after receiving a prompt that the participant had completed the questionnaire. The researcher ensured that the questionnaire was complete and correctly submitted by the participant. Each participant was allowed to respond only once, and questionnaires with an answer time of less than 180 s or with identical choices were considered invalid. Participants were assured that their data would remain anonymous and confidential. The collected data were stored securely on a disk, accessible only to the study researchers, and strict confidentiality was maintained. After the completion of the questionnaire, the researcher checked for any omissions and collected the questionnaires.

### Statistical analysis

Statistical analysis was conducted using IBM SPSS software, version 21.0, and SPSS-AMOS 24.0 (IBM Corporation, Chicago, IL, USA). The variables meet the normality assumption. Descriptive statistics, including means, standard deviations (SD), frequencies, and percentages, were used to summarize continuous and categorical variables. Pearson correlation analysis was performed to examine the relationships between variables. Exploratory factor analysis was employed to mitigate common method biases.

SEM was utilized to construct and assess the chain mediating model. The significance of the mediating effect was determined using the bias-corrected bootstrapping method^24^. Full mediation was confirmed if the direct effect was nonsignificant, whereas partial mediation was confirmed if the direct effect was significant. Model fit was evaluated using several indices, including the Ratio of Chi-Square to Degrees of Freedom (χ^2^/df)^24^, Root Mean Square Error of Approximation (RMSEA)^25^, Adjusted Goodness of Fit Index (AGFI)^26^, Goodness of Fit Index (GFI)^27^, Comparative Fit Index (CFI)^27^, and Tucker-Lewis Index (TFI)^27^. Adequate fit was indicated by AGFI, GFI, CFI, and TFI values greater than 0.90, an RMSEA value less than 0.08, and a χ^2^/df value less than 3. The statistical tests conducted in this study were two-tailed, and a significance level of P < 0.05 was deemed as statistically significant.

### Ethical approval

Before being permitted to participate in the study, participants were informed of the purpose of the research, the meaning and data security. In addition, participation was voluntary and anonymous, they were informed of their rights and responsibilities and that they had the right to withdraw from participation at any time. This study was approved by the ethics committee of Renmin Hospital of Wuhan University (No: WDRY2022-K192). All participants gave their voluntary written informed consent prior to study participation.

## Results

### Common method *bias* test

To assess common method biases in this study, exploratory factor analysis was conducted due to the use of self-reported data. The results revealed that 9 variables had characteristic roots greater than 1. However, the first factor accounted for only 26.30% of the total variance, which was less than the recommended threshold of 40%^28^. These findings suggest that there were no significant issues related to common method biases in this study.

### Participant characteristics

Out of the 227 participants, 56.80% were male. The mean age was 54.15 (SD = 15.12) years, ranging from 20 to 93 years. Among the participants, 28.60% had an education level higher than high school, 78.00% were married, 51.10% had undergone dialysis for more than 3 years, 60.40% had chosen autogenous arteriovenous fistula, and 40.10% had diabetes. Further details are presented in Table 1.

**Table 1:** Characteristics of the participants (*N* = 227).

Variables	Categories	N (%)	Range	Mean ± SD
Gender	Male	129(56.80)	–	–
	Female	98(43.20)	–	–
Age	< 45	72(31.72)	20 ~ 44	35.75 ± 6.61
	45 ~ 60	71(31.28)	47 ~ 60	54.80 ± 3.88
	> 60	84(37.00)	61 ~ 93	69.38 ± 6.58
Education	Primary	63(27.80)	–	–
	Secondary	99(43.60)	–	–
	Higher	65(28.60)	–	–
Marital status	Married	177(78.00)	–	–
	Single	38(16.70)	–	–
	Divorced∕Widowed	12(5.30)	–	–
Duration of dialysis (month)	< 12	55(24.20)	4 ~ 11	8.60 ± 2.11
	12 ~ 36	56(24.70)	12 ~ 36	21.79 ± 6.81
	> 36	116(51.10)	37 ~ 280	89.78 ± 43.86
Type of vascular access	Autogenous arteriovenous fistula	137(60.40)	–	–
	Artificial blood vessels	16(7.00)	–	–
	Central venous catheter	74(32.60)	–	–
Diabetes	Yes	91(40.10)	–	–
	No	136(59.90)	–	–

The Sociodemographic characteristics form was sourced from references 7 to 10.

### Anxiety, sleep quality, hope and family function scores

The mean anxiety score was 4.36 (SD = 4.02), with a prevalence rate of 35.24% (80 cases). The mean sleep quality score was 7.57 (SD = 4.74), and the prevalence rate was 81.01% (suspected insomnia: 79 cases; insomnia: 113 cases), indicating a high prevalence of sleep disorders in patients receiving MHD. The mean hope score was 34.64 (SD = 7.21), indicating a moderate level of hope. The mean family function score was 6.21 (SD = 2.90), indicating moderate impairment. Further details can be found in Table 2.

**Table 2:** Anxiety, sleep quality, hope and family function scores (*N* = 227).

Variables	Number of items	Range of score	Score	Equalization of items	Prevalence rate
Anxiety	7	0 ~ 21	4.36 ± 4.02	0.62 ± 0.57	35.24%
Sleep quality	8	0 ~ 24	7.57 ± 4.74	0.95 ± 0.59	81.01%
Hope	12	12 ~ 48	34.64 ± 7.21	2.89 ± 0.60	-
Temporality and future	4	4 ~ 16	11.73 ± 2.45	2.93 ± 0.61	-
Positive readiness and expectancy	4	4 ~ 16	11.55 ± 2.89	2.89 ± 0.72	-
Interconnectedness	4	4 ~ 16	11.37 ± 2.66	2.84 ± 0.67	-
Family function	5	0 ~ 10	6.21 ± 2.90	1.24 ± 0.58	-
Adaptation	1	0 ~ 2	1.33 ± 0.76	1.33 ± 0.49	-
Partnership	1	0 ~ 2	1.17 ± 0.79	1.17 ± 0.79	-
Growth	1	0 ~ 2	1.15 ± 0.81	1.15 ± 0.81	-
Affection	1	0 ~ 2	1.12 ± 0.78	1.12 ± 0.78	-
Resolve	1	0 ~ 2	1.28 ± 0.84	1.28 ± 0.84	-

### Correlation analysis of anxiety, sleep quality, hope and family function

Pearson correlation analysis revealed significant associations among anxiety, sleep quality, hope, and family function. Anxiety was negatively correlated with hope and family function (r = − 0.314, − 0.475, P < 0.01, respectively), while it was positively correlated with sleep quality score (r = 0.477, P < 0.01). Hope was positively correlated with family function (r = 0.467, P < 0.01), while sleep quality score was negatively correlated with hope and family function (r = − 0.585, − 0.652, P < 0.01, respectively). Further details can be found in Table 3.

**Table 3:** Correlations between observed variables (*r*).

Variable	1	2	3	4	5	6	7	8	9	10	11	12
1 Anxiety	1.000											
2 Sleep quality	0.477**	1.000										
3 Hope	− 0.314**	− 0.585**	1.000									
4 Temporality and future	− 0.272**	− 0.488**	0.874**	1.000								
5 Positive readiness and expectancy	− 0.297**	− 0.574**	0.929**	0.730**	1.000							
6 Interconnectedness	− 0.277**	− 0.513**	0.896**	0.655**	0.759**	1.000						
7 Family function	− 0.475**	− 0.652**	0.467**	0.392**	0.468**	0.396*	1.000					
8 Adaptation	− 0.201**	− 0.405**	0.242**	0.166*	0.233**	0.251*	0.651**	1.000				
9 Partnership	− 0.206**	− 0.402**	0.387**	0.307**	0.385**	0.349**	0.612**	0.239**	1.000			
10 Growth	− 0.352**	− 0.451**	0.303**	0.225**	0.316**	0.271**	0.704**	0.471**	0.286**	1.000		
11 Affection	− 0.372**	− 0.462**	0.441**	0.422**	0.405**	0.369**	0.693**	0.302**	0.462**	0.382**	1.000	
12 Resolve	− 0.472**	− 0.533**	0.267**	0.219**	0.282**	0.216**	0.737**	0.439**	0.306**	0.533**	0.442**	1.000

*P < 0.05; **P < 0.01.

### Mediating effect analysis

To test the research hypotheses, a SEM was employed, which included anxiety as the independent variable, hope and family function as mediating variables, and sleep quality as the dependent variable. Anxiety and sleep quality were observed variables, while hope and family function were latent variables. Factors 1, 2, and 3 were used as indicators of hope, and adaptation, partnership, growth, affection, and resolve were used as indicators of family function. After modification, the model fit indices were as follows: χ^2^/df = 1.757, RMSEA = 0.058, GFI = 0.954, AGFI = 0.915, CFI = 0.976, and TLI = 0.976. These indices indicated a good fit for the model.

**Figure 2:**
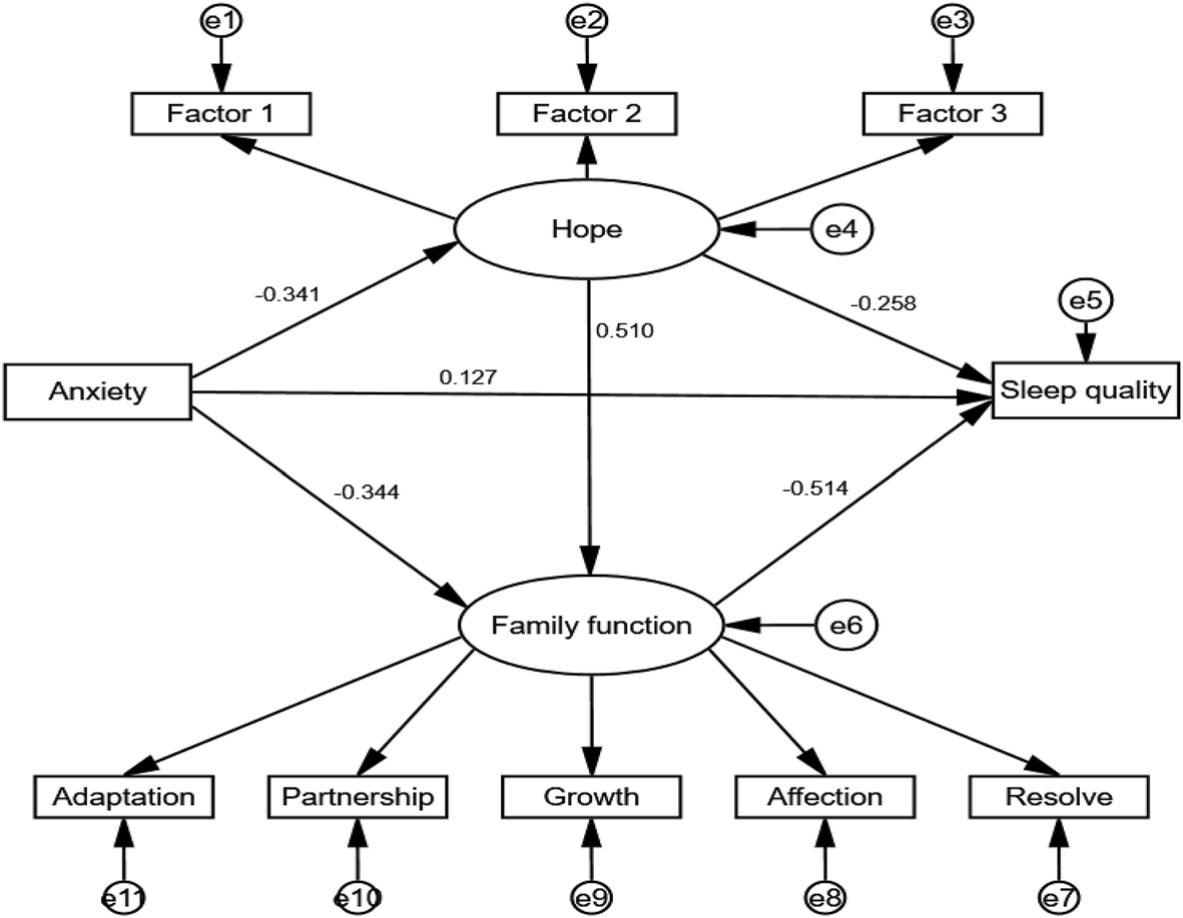
Finalized structural model (*N* = 227). Note: Factor loadings are standardized.

Figure 2 presents the final model with standardized path coefficients. The results showed that anxiety positively predicted sleep quality score (β = 0.127, P = 0.001), supporting hypothesis H1. Furthermore, anxiety had a negative effect on hope (β = -0.341, P = 0.001) and family function (β = -0.344, P = 0.001). Hope positively predicted family function (β = 0.510, P = 0.001), and both hope (β = -0.258, P = 0.001) and family function (β = -0.514, P = 0.001) had negative effects on sleep quality score.

To test the mediating effects of hope and family function, bias-corrected bootstrapping with 5000 iterations was conducted. The 95% bootstrap confidence intervals (CI) were used to determine the significance of the mediating effects. The results indicated that the 95% CI (0.275 to 0.442) of the total indirect effect between hope and family function on anxiety and sleep quality did not include 0, suggesting a significant mediating effect. Additionally, the 95% CI (0.006 to 0.246) of the direct effect of anxiety on sleep quality did not include 0, indicating a significant direct effect, supporting H1.

Furthermore, the 95% CI (0.023 to 0.159) of the indirect effect 1 between hope and anxiety and sleep quality, the 95% CI (0.108 to 0.275) of the indirect effect 2 between family function and anxiety and sleep quality, and the 95% CI (0.045 to 0.172) of the indirect effect 3 between hope, family function, and anxiety and sleep quality did not include 0, indicating significant mediating effects. These results support H2, H3, and H4. Therefore, the types of mediation in this study were identified as partial mediation.

The direct effect value of anxiety on sleep quality was 0.127, the total indirect effect value was 0.354, the total effect value was 0.481, and the total effect size was 73.60% (0.354/0.481). Detailed results can be found in Table 4.

**Table 4:** Total, direct, total indirect and specific indirect effects (*N* = 227).

Structural path	Standard coefficients (Effect value∕β)	*95% CI*		Effect size(%)
		Lower	Upper	
Total effect	0.481	0.373	0.579	100.00
Direct effect	0.127	0.006	0.246	26.40
Total indirect effect	0.354	0.275	0.442	73.60
Indirect effect 1	0.088	0.023	0.159	18.30
Indirect effect 2	0.177	0.108	0.275	36.80
Indirect effect 3	0.089	0.045	0.172	18.50

Indirect effect 1: Anxiety → Hope → Sleep quality.

Indirect effect 2: Anxiety → Family function → Sleep quality.

Indirect effect 3: Anxiety → Hope → Family function → Sleep quality.

## Discussion

The results of this study indicate that patients receiving MHD experience a certain degree of anxiety, while hope and family function are at a moderate level. However, sleep quality disorders are found to be more severe among these patients. There is a significant correlation among all variables, and hope and family function play a chain mediating role between anxiety and sleep quality.

Several factors contribute to the serious sleep quality disorders observed in this study. Firstly, 91 MHD patients in this study have comorbid diabetes, which has been shown to induce multiple organ damage, disrupt the transmission of neurotransmitters in the central nervous system, and lead to autonomic nervous disorders, resulting in various sleep disorders^29^. Secondly, the average age of the study subjects is 54.15 years (SD = 15.12; range from 20 to 93), which is relatively old. Age has been identified as an independent risk factor for sleep disorders^30^. Thirdly, MHD patients generally have compromised immune systems and frequently visit hospitals, which increases the risk of infection and psychological burden, further exacerbating sleep disorders^31^. Fourthly, symptom burden also negatively affects sleep quality.

### Anxiety can directly predict sleep quality (Direct effect: Anxiety → Sleep quality)

The results of this study reveal that anxiety has a direct and negative impact on sleep quality in patients undergoing MHD^10^. This implies that higher levels of anxiety are associated with poorer sleep quality. Individuals with higher anxiety sensitivity may be more prone to experiencing negative thoughts and emotions, which can activate a neuroendocrine response and ultimately lead to decreased sleep quality^32^. In essence, anxiety sensitivity may influence an individual's sleep quality through the cognitive bias pathway, involving automatic thinking patterns^32^.

### Hope plays a partial mediating role between anxiety and sleep quality (Indirect effect 1: Anxiety → Hope → Sleep quality)

The findings of this study indicate that hope acts as a partial mediator between anxiety and sleep quality in patients undergoing MHD. This result provides theoretical evidence that anxiety can influence sleep quality through the mediating effect of hope in this population. There exists a robust correlation between anxiety and hope, supported by evidence that positive emotions can boost patient´s self-efficacy in confronting their illness, thus heightening their level of hope^33^. As a protective factor for sleep quality, hope can facilitate patients' positive cognitive appraisal of their illness, their evaluation of its significance, and their willingness to make changes in their attitudes and lifestyle^34^. This, in turn, builds their confidence and courage to cope with their illness, ultimately leading to improvements in sleep quality.

### Family function plays a partial mediating role between anxiety and sleep quality (Indirect effect 2: Anxiety → Family function → Sleep quality)

The findings of this study demonstrate that family function acts as a partial mediator between anxiety and sleep quality in patients undergoing MHD. This result provides theoretical evidence that anxiety can influence sleep quality through the mediating effect of family function in this population. Patients with lower levels of anxiety tend to have better communication and express their inner feelings more effectively to their family members, leading to a more active and functional family dynamic, which significantly reduces the occurrence of family dysfunction^35^. Family function serves as a protective factor in improving sleep quality. The support and understanding received from family members can help alleviate patients' perceived stress, ultimately leading to improvements in sleep quality. This finding aligns with the principles of sleep quality in Traditional Chinese Medicine (TCM), which emphasize the importance of alleviating negative emotions, stabilizing physiological functions, promoting the balance of vital energy and blood, and cultivating a peaceful mind to improve sleep disorders and promote overall well-being^13^.

### Hope and family function play a chain mediating effect in the relationship between anxiety and sleep quality (Indirect effect 3: Anxiety → Hope → Family function → Sleep quality)

The results of this study indicate that hope and family function have a chain mediating effect in the relationship between anxiety and sleep quality in patients undergoing MHD. This suggests that anxiety can enhance family function by promoting hope, thereby improving sleep quality. Lower levels of anxiety are associated with increased levels of hope in MHD patients. Patients with higher levels of hope exhibit greater trust in their social support network, actively seek assistance, and receive support from family members and other sources, leading to improved family communication, partnership, and affection^36^. The improvement in family function, in turn, significantly enhances sleep quality in these patients.

The implications of this study for clinical nursing practice are significant. By utilizing the SEM approach, we have shed light on the chain mediating effect of hope and family function in the relationship between anxiety and sleep quality. These findings emphasize the importance of considering anxiety, hope, and family function as crucial factors in addressing sleep quality issues. Based on these results, the following intervention measures can be recommended to improve sleep quality in MHD patients.

Firstly, healthcare professionals can employ cognitive-behavioral therapy, mindfulness therapy, sensory art therapies, and other psychotherapeutic methods to alleviate anxiety symptoms^37–40^. TCM approaches, such as the five-element music therapy and Ba-duan-jin exercise, have also shown effectiveness in reducing anxiety^41,42^.

Secondly, healthcare professionals can assist patients in developing self-management strategies, promoting positive coping styles, and implementing effective symptom management measures to enhance the level of hope^43–45^.

Lastly, family system therapy, as well as ecosystem-focused therapy, continuity care, chain family care, and hospital-community-family care interventions, can be utilized to improve family functioning in MHD patients^46–48^. These approaches focus on enhancing family dynamics and support, which can have a positive impact on sleep quality.

## Limitations

Notwithstanding the notable outcomes of this investigation, it is imperative to acknowledge several constraints that should be taken into account.

Firstly, the data collection was limited to two tertiary hospitals in Wuhan, may limiting the generalizability of the results to other geographical areas within China. Conducting multi-center studies with larger sample sizes would help to validate the model and enhance the generalizability of the results. Future research should aim to include a more diverse sample from multiple regions across China.

Secondly, the use of self-reported questionnaires introduces a subjective element to the data, which may not fully capture the actual experiences of the patients. To obtain a more comprehensive and accurate evaluation of sleep quality, it would be beneficial to incorporate both subjective and objective assessment tools. This could include integrating biochemical examinations and medical history information from electronic systems to provide a more holistic assessment of sleep quality.

Thirdly, this study did not explore the longitudinal and in-depth mechanisms underlying the influence of anxiety, hope, and family function on sleep quality. Further research should employ longitudinal designs and investigate additional variables to better understand the complex relationships and mechanisms involved. Long-term follow-up studies would provide valuable insights into the dynamic nature of sleep quality in this population.

## Conclusion

In conclusion, this study establishes the chain mediating effect of hope and family function between anxiety and sleep quality in patients undergoing maintenance MHD. The findings highlight the importance of addressing anxiety and promoting hope and family function to improve sleep quality in this population. Healthcare professionals should implement targeted interventions to help patients alleviate anxiety, enhance hope, and improve family functioning.

It is noteworthy that the present investigation was conducted solely in China, and such, one must exercise prudence when extrapolating the findings to other nations, given the potential cultural variances between China and the Western context. Consequently, it would be advantageous to corroborate the outcomes of this study in MHD cohorts from diverse nations, while also acknowledging the cultural heterogeneity, in forthcoming research efforts.

## Data Availability

The data that support the findings of this study are available from the corresponding author upon reasonable request. The data sets generated during or analyzed during the current study are not publicly available due to the subject confidential information.

## Abbreviations

CKD: Chronic kidney disease
ERSD: End-stage renal disease
MHD: Maintenance hemodialysis
SEM: Structural equation modeling
HADS: Hospital anxiety and depression scale
AIS: Athens insomnia scale
HHI: Herth hope index
APGAR: Adaptation, partnership, growth, affection, and resolve
χ2/df: Chi-square to degrees of freedom
RMSEA: Root mean square error of approximation
AGFI: Adjusted goodness of fit index
GFI: Goodness of fit index
CFI: Comparative fit index
TFI: Tucker-Lewis index
SD: Standard deviation
CI: Confidence intervals
TCM: Traditional Chinese medicine
